# Is Opium-Eating Injurious?

**Published:** 1881-12

**Authors:** 


### IS OPIUM-EATING INJURIOUS?

Mr. D. H. Cullimore, writing to the *Br. Med. Jour.* of June 25, in reply to the query of Dr. Murrell, "Is opium-eating and opium-smoking necessarily and universally pernicious?" says: "In one respect it is more pernicious than either tobacco or alcohol, and that is, in the fact that its use is more liable to degenerate into abuse than either of these. In moderation, I have been told, many people look upon their daily supply as a fillip—a sherry-and-bitters, in fact, and in numerous instances it has been found, after a lifetime, as little injurious. On the other hand, when the mind has become unbalanced from pecuniary loss or domestic calamities, the usual allowance is exceeded, and what was originally a moderate dose would eventually be looked upon as a homœopathic or even an infinitesimal one. Moderation, even here, is often regulated by the purchasing power of the individual; and this, as the drug is expensive, will ever remain a barrier, not only against its general abuse, but even against its universal use, amongst the immense population of China. Thus, in Singapore, where the wages of the Chinese are good, the consumption of opium is at the rate of 330 grains a year for each person. In Java, where the Chinese community is a small one, and the wages comparatively low, the consumption does not exceed 40 grains; and in China itself, the rate of consumption is 140 grains for adult doses, which would not give half a grain a day. But, as I said before, it is inaccessible to the mass of the people, owing to their poverty. In many cases with which I was familiar, it did not produce constipation or loss of appetite, even when attended with listlessness and voluptuous reveries. No such effects followed as the result of separate doses; but, as a rule, its continued use will eventually end in loss of appetite, constipation, mental decay, and sexual impotency. The classic description of what I should call the confirmed rather than the habitual opium-eater, quoted by Dr. Murrell, though exaggerated and highly colored—as such descriptions are likely to be—is in many particulars correct. For instance, a casual observer can recognize at a glance among any collection of well-to-do Chinamen assembled round the joss-house, or other resort, a few possessing most distinctive characters of the confirmed debauchee. I have never seen a death the immediate and exciting cause of which was opium-eating, but can remember a few the result of an active and excessive debauch with this drug in conjunction with bang, an extract of Indian hemp."—*N. Y. Med. Times.*

